# Nurses’ Experiences as Care Providers for Monolingual Patients: Insight and Suggestions for Communication During Nursing Care in Multicultural Societies

**DOI:** 10.1177/10436596251366869

**Published:** 2025-09-12

**Authors:** Johannes Mwapotelange, Vistolina Nuuyoma

**Affiliations:** 1 University of Namibia, Windhoek, Namibia

**Keywords:** communication and language, monolingual patients, multilingual society, nursing practice, patient care

## Abstract

**Introduction:**

The language barrier may cause ineffective communication during nursing care, and its potential consequences are felt in multicultural and multilingual societies due to language diversity. This study explored nurses’ experiences of providing nursing care to monolingual patients at a national referral hospital in Namibia.

**Methods:**

This qualitative, explorative, and descriptive research was conducted with conveniently sampled nurses who were then interviewed individually. These nurses worked for over a year and could speak only two or less languages. Data analysis followed Braun and Clarke’s six phases of reflexive thematic analysis.

**Results:**

From interviews with 19 nurses, four main themes were revealed: personal benefits, negative experiences, alternative communication approaches, and strategies to improve nursing care for monolingual patients.

**Discussion:**

These results have implications for providing culturally congruent care by revealing the importance of recruiting professional interpreters and multilingual nurses, incorporating language courses into undergraduate curricula, and providing in-service training for nurses.

## Introduction

Language is key to effective communication between nurses and patients, and is paramount in providing all health care services (Kwame & Petrucka, 2021). Barriers to effective communication are multifaceted, including work conditions, organizational factors, and a lack of mutual understanding and awareness. Other barriers relate to emotional and psychological distress, as well as the language barrier between nurses and patients (Gheshlagh et al., 2024). Language barriers, specifically in health care settings, reduce providers’ and patients’ satisfaction, decrease care provision quality, and compromise patient safety (Al Shamsi et al., 2020). The impacts of language barriers on patients include poor health-seeking behavior, occurrences of preventable medical errors, poor adherence to treatment, increased length of hospital stays, weak therapeutic relations, and delayed treatment due to misdiagnosis (de Moissac & Bowen, 2019; Olani et al., 2023). These negatively affect patients’ health outcomes by increasing disease morbidity and mortality.

Language barriers and their potential consequences are felt in multicultural and multilingual societies due to the diversity of languages (Osae-Larbi, 2016). Within these societies, there are multilingual, bilingual, and monolingual people. Monolingualism is the state of people who have adopted the grammar of only one linguistic system that has the complexity of a human language (Jouitteau, 2021). This led to the ability to speak only a single language (Küçükler & Tosuncuoglu, 2018). Monolingual patients cannot effectively communicate their symptoms and health concerns to health care workers who speak a different language, thus increasing the possibility of not receiving needed care. Moreover, there is a possibility of misunderstandings, errors, and a lack of informed consent to nursing interventions (Alamri et al., 2021). Paredath et al. (2023) associated monolingualism with misunderstandings of patients’ basic requests and the inability of nurses to explain procedures and health facility guidelines to patients and update them on their conditions. This indicates an urgent need to understand how nurses care for monolingual patients.

Nurses are important in establishing and maintaining patient communication (Tetteh et al., 2021). However, this becomes difficult when dealing with monolingual patients who speak different languages from the nurses. To the best of our knowledge, there are limited studies on nurses’ reflections on their experiences of rendering nursing care to monolingual patients, and this has not been investigated in Namibia or other similar under-resourced and multicultural societies. A previous study conducted in Namibia focused on the medical doctors’ perspectives on using the English language (Basimike, 2018). Mlambo (2017) investigated the communicative practices of multilingual health practitioners. While Ashipala and Matundu (2023) focused on nursing students’ experiences in Namibia’s multilingual and multicultural clinical environment. Namibia is considered a multicultural and multilingual society, with about 13 ethnic groups associated with diverse native languages. Ethnic groups in Namibia include the Aawambo, vaKavango, Hereros, Nama, Damara, San people, Tswana, Rehoboth Basters, people of mixed race, Caprivians, White Namibians, mainly Afrikaners, and others of German, British, and Portuguese ancestry. In addition, there are Ovahimba people who live a pastoral and traditional semi-nomadic lifestyle. The official language of Namibia is English; however, not everyone can communicate in English, particularly older individuals and those from remote areas of the country. Therefore, our study aimed to explore nurses’ experiences of providing nursing care to monolingual patients at a national referral hospital in Namibia.

## Materials and Methods

### Ethical Considerations

This study has received ethical clearance from the School of Nursing and Public Health Ethics Committee at the University of Namibia (campus level) and written permission from the Ministry of Health and Social Services. Additionally, written permission was obtained from the Office of the Chief Medical Officer of the referral hospital. The supervisors of various hospital sections granted oral permission. Participants signed consent forms for their participation and audio recordings and were informed of their right to withdraw from the study at any time.

### Study Design

This qualitative study was designed as an explorative and descriptive research. These designs helped observe and explore the dynamic and context-dependent notion of realities, meanings, knowledge, and the social world (Phillips, 2023). This was to answer the main research question: “What are nurses’ experiences as care providers for monolingual patients at a national referral hospital in Namibia?” The Consolidated Criteria for Reporting Qualitative Research (COREQ) was followed as a reporting guideline (Tong et al., 2007).

### Setting and Sample

The setting was a national referral hospital in Windhoek, the capital of Namibia. This hospital has a capacity of 1,101 beds, with 582 nursing staff. Using convenience sampling, nurses were recruited by posting flyers with study information on hospital WhatsApp groups and sharing them across different hospital units. Invited to participate in the study were (a) nurses who had worked for more than 1 year, (b) who were only able to speak two languages or less, which is English and their mother tongue or English only, and (c) able and willing to be interviewed. Exclusion criteria were (a) nurses working in nonclinical units and (b) no direct involvement in patient care. Nineteen nurses participated in the study, a sample determined by data saturation. Table 1 displays the participants’ demographic characteristics.

**Table 1.** Demographic Characteristics of Participants.

Participant’s Code	Age (in years)	Gender	Qualifications	Years of Nursing Work Experience	Hospital Department	Mother Tongue	Number of Languages Spoken^a^
N1	26	F	BNS^b^	3	Antennal ward	Oshiwambo	2
N2	32	F	CNM^c^	6	Outpatient department	Rukwangali	2
N3	24	F	BNS	2	Antennal ward	Oshiwambo	2
N4	25	F	BNS	3	Stroke unit	Orudhemba	2
N5	29	F	BNS	3	Surgery ward(3B)	Oshiwambo	2
N6	35	F	CNM	2	Orthopedic ward	Oshiwambo	2
N7	30	F	BNS	5	Respiratory Unit	Subia	2
N8	41	F	CNM	7	Stroke Unit	Oshiwambo	2
N9	29	F	BNS	6	Medical Ward	Oshiwambo	2
N10	34	F	BNS	5	Respiratory Unit	Silozi	2
N11	31	F	CNM	3	Medical ward (5A)	Afrikaans	2
N12	32	F	BNS	2	Stroke Unit	Oshiwambo	2
N13	29	F	BNS	4	Post Natal ward	Afrikaans	2
N14	32	F	BNS	3	Maternity ward	Oshiwambo	2
N15	28	F	BNS	5	Prem Unit	Oshiwambo	2
N16	26	F	CNM	2	Surgical ward	Silozi	2
N17	29	M	CNM	3	Head Injury Unit	Oshiwambo	2
N18	26	M	CNM	2	Surgical ward	Silozi	2
N19	26	M	BNS	4	Orthopedic ward	Rukwangali	2

a Counting mother tongue/native language and English. ^b^ Bachelor of Nursing Science. ^c^ Certificate in Nursing and Midwifery Science.

### Data Collection

Data were collected from May to September 2024. Interested nurses who met the inclusion criteria scheduled the interviews at their convenience time and venue. Data were collected through individual face-to-face interviews conducted using an interview guide. The authors developed an interview guide and piloted it with four nurses before the main data collection to ensure practical feasibility and identify flaws (Malmqvist et al., 2019). No participant dropped out of the study, and no follow-up interviews were conducted. The boardroom was the preferred venue for most participants, and it was quiet with minimal interruptions. The duration of the interviews was between 30 and 45 min, all of which were audio recorded. Interviews were conducted until data saturation, when new participants started repeating what previous ones had stated. Further evidence was the absence of new codes or themes emerging during the analysis of transcripts, implying that the data were saturated (Saunders et al., 2018). Field notes were taken to write down nonverbal communication and researchers’ reflections on the research process.

### Data Analysis

Audio recordings from the interviews were stored in a password-protected mobile device, transferred to a personal computer, and transcribed verbatim with the cloud-based Word program under the Microsoft 365 software suite. The two authors analyzed data manually; no qualitative data analysis software was used. The first author performed initial coding and then met the second author for presentation, further analysis, and consensus on themes and subthemes. Data were analyzed according to the six phases of reflexive thematic analysis (Braun & Clarke, 2021), suitable for explorative and descriptive research. Table 2 shows the phases followed during data analysis.

**Table 2.** Braun and Clarke’s Six Phases of Reflexive Thematic Analysis and Application to the Study.

Phase	Application to the study
**Phase 1:** Familiarization and writing notes	The first author read through transcriptions several times while making notes of key aspects identified as experiences.
**Phase 2:** Systematic data coding	The first author identified codes by highlighting key concepts, and phrases to use them to come up with codes.
**Phase 3:** Generating initial themes from coded and collated data	Two authors met for the presentation of codes, and then grouped similar codes and came up with subthemes.
**Phase 4:** Developing and reviewing themes	Similar subthemes were grouped to form themes. It is at this phase where a coding tree was designed (Figure 1).
**Phase 5:** Refining, defining, and naming themes	The two authors had a meeting to reach a consensus on the naming of themes that were taken as findings of the study.
**Phase 6:** Writing report	The first author drafted the report of findings, under supervision, and edited by the second author.

### Rigor

This study followed recommendations for strategies for establishing rigor in qualitative inquiry (Morse, 2015). Table 3 displays how these strategies were applied to the study.

**Table 3.** Strategies Used in the Study to Establish Rigor.

Strategies	Application to the study
Prolonged engagement	Spend adequate time in the field (May–September 2024) in order to build rapport with participants and allow for data saturation.
Thick descriptions	Detailed descriptions of research methods and motivation of choices.
Peer debriefing	Engaged with peers to seek feedback and validation
Triangulation of participants	Participants from different cultural groups, and different native languages Recruitment of participants from different units such as maternity, outpatient department, pediatric, outpatient, surgical, medical, surgical, and orthopedics wards.
Development of a coding system	Following reflexive thematic phases and the use of two coders.
Member checking	Shared transcripts with participants
Pilot testing	The interview guide pilot tested with four participants who met the inclusion criteria.
Reflexivity	Researchers’ reflections on the research process by making notes in the research diary and field notes. Researchers noted personal biases and preconceptions of monolingual patients and the research processes involved.

## Results

**Figure 1.**
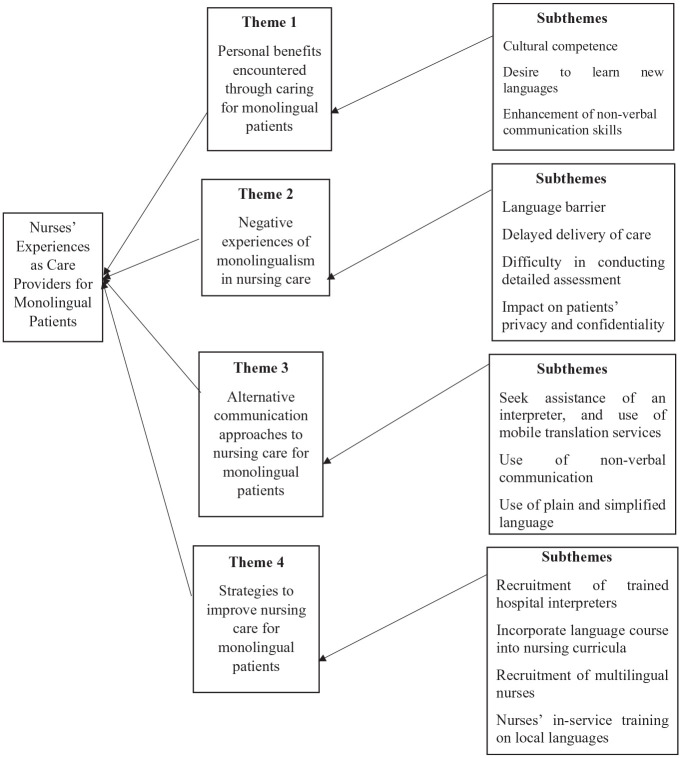
Coding Tree for Nurses’ Experiences as Care Providers for Monolingual Patients

Four themes emerged from the analysis: (1) personal benefits encountered through caring for monolingual patients; (2) negative experiences of monolingualism in nursing care; (3) alternative communication approaches to nursing care for monolingual patients, and (4) strategies to improve nursing care for monolingual patients. Figure 1 outlines the themes and their subthemes as a coding tree.

### Theme 1: Personal Benefits Encountered Through Caring for Monolingual Patients

Nurses benefit by developing cultural competence and a desire to learn different languages. In addition, monolingual patients assisted nurses in developing interpreting and communicating abilities via nonverbal techniques.

#### Subtheme 1.1: Cultural Competence

Development of cultural competence was facilitated by the appreciation of different cultures and languages during nurses’ and patients’ interactions, which assisted in nurturing respect for other cultures. This way, nurses address and care for patients in a culturally acceptable manner, building trust and better communication. Participant 4 said this about cultural awareness: “It gave me a deeper understanding, and I was able to appreciate different cultures and languages” **N4**.

#### Subtheme 1.2: Desire to Learn New Languages

On the other hand, a strong desire to learn new languages emerged as a benefit for nurses who provide nursing care to monolingual patients. Participant 9 mentioned, “it gave me an opportunity and desire to learn other people’s language” **N9**. Participant 12 added, “It motivates me to try and learn different languages” **N12**.

#### Subtheme 1.3: Enhancement of Nonverbal Communication Skills

Moreover, nurses reported that the need to communicate effectively with monolingual patients led them to develop interpreting and communicating abilities via nonverbal communication strategies. Participant 4 mentioned how providing care to monolingual patients enhanced their nonverbal communication skills; “So, it taught me many things, which helped me develop strong nonverbal communication skills” **N4**. Participant 1 added, “I used body language and sign language to ask her how she feels. She managed to show me at least that she was cut there once, like operated once, and now she is in pain” **N1.**

### Theme 2: Negative Experiences of Monolingualism on Nursing Care

Negative experiences were reported concerning language barriers between a nurse and a patient and delayed care provision due to challenges in conducting thorough assessments. Moreover, it was difficult to maintain privacy and confidentiality when dealing with a monolingual patient.

#### Subtheme 2.1: Language Barrier

Nurses expressed that it was difficult to communicate during nursing care provision since patients only spoke one language. Participant 13 expressed, “I could not understand because this patient could only speak one language” **N13**. Participant 9 said, “I remember the diabetic patients who were hospitalised, and later they were discharged. I explained everything to them, like how and when to take medications. They ended up taking more medication, which resulted in the medication being finished before their next pharmacy follow-up” **N9**.

#### Subtheme 2.2: Delayed Delivery of Care

Nurses revealed that the inability to communicate effectively with monolingual patients often delays care delivery. This is because additional time was spent trying to translate or wait for interpreters, ultimately prolonging nursing care processes. The delay may negatively affect patient outcomes, particularly in acute care settings. Participant 12 expressed this on delayed care services delivery; “I went to look for someone to interpret, and everything was delayed” **N12**. Participant 3 added, “it was hard and time-consuming for me to make this patient understand since they only spoke one language” **N3**.

#### Subtheme 2.3: Difficulty Conducting a Detailed Assessment

Since patients only spoke one language, nurses failed to comprehend their health concerns and thus could not conduct proper assessments to make informed nursing diagnoses. This leads to nursing care plans unrelated to patients’ health concerns. Participant 2 mentioned this, “I could not interpret their complaints, so I plan for something else that is not what is happening” **N2**.

#### Subtheme 2.4: Impact on Patients’ Privacy and Confidentiality

In seeking to understand patients’ health concerns properly, nurses sought people to interpret during their communication with patients; these people are referred to as *ad hoc* interpreters. An *ad hoc* interpreter is any available person who speaks the patient’s language, such as a family member, friend, bilingual, or multilingual staff member (Probst & Imhof, 2016). People commonly used as “ad hoc interpreters” are nursing students, nurses, doctors, family members, and others available in health care facilities who speak the patients’ languages. However, involving others, especially nonhealth care professionals, made nurses concerned about patients’ privacy and keeping their health information confidential. To adhere to privacy and confidentiality, nurses preferred to use other health professionals and online language applications for interpretation. This was revealed by Participant 1: “I try to avoid including people that are outside the health care systems because they do not know how to maintain confidentiality. So, I must ensure that my interpreter is from the health-related field” **N1**. Participant 3 added, “I look for a professional staff member because they all understand the importance of privacy and confidentiality. If there is no staff member, I would take a student and not another patient, so they do not get information on other patients” **N3**.

### Theme 3: Alternative Communication Approaches to Nursing Care for Monolingual Patients

To communicate with monolingual patients, nurses used various communication approaches, including seeking assistance from translators, mobile translation services, sign language, and simplified language.

#### Subtheme 3.1: Seek the Assistance of an Interpreter and Use Mobile Translation Services

Nurses’ willingness to effectively communicate with patients led to them resorting to *ad hoc* interpreters and mobile translation services. The interpretation was made by the patient’s relative, who spoke and understood English; even though not in their company, the patient called a relative and had them interpret telephonically. Participant 2 mentioned this, “When working with a patient, he or she can call a relative, put on a phone speaker, then communicate through that third person” **N2**. Participant 18 added, “Just put them on the speaker; that way, the patient felt at ease speaking to someone who understands their language” **N18**. Participant 1 stated, “Sometimes I involve the family; you know it is a team, right?” **N1**. Participant 3 described how online translation services were used; “I use my phone to Google how to say something in a language the patient speaks, and I give them the phone so they type what they want to reply to me” **N3**.

Moreover, different live applications on mobile phones and recorded patient education sessions in a language understood by the patient were used as alternative communication approaches. Participant 6 said, “I use my phone to explain post-operative care instructions and the potential risks; I have them saved in different languages” **N6**. Sometimes, patients try to communicate with nurses using mobile applications and artificial intelligence services on their cell phones. Participant 3 described this, “I have seen patients who have apps on their phones, so when you say hello, their app will respond to or translate for them” **N3**.

#### Subtheme 3.2: Use of Nonverbal Communication

When effective communication was impossible due to a language barrier, nurses indicated they communicated with patients nonverbally. This was necessary for obtaining crucial information about patient needs. Participant 1 said, “The patient could not communicate in English or Oshiwambo. So, I used nonverbal signs to ask her how she was feeling; she showed me using sign language”: **N1**. Participant 2 added, “I nod and use my hands to communicate” **N2**.

#### Subtheme 3.3: Use of Plain and Simplified Language

Nurses emphasized the importance of using plain and simplified language to ensure patients’ understanding by avoiding jargon and complex terminology. They supported communication with shorter sentences and simple language, making it easier for patients to comprehend medical and nursing instructions. Participant 3 shared this experience: “I try by all means to express myself with a few words slowly and using simple language that is straightforward to what I want to communicate. I avoid jargon words or complex terminologies” **N3**.

### Theme 4: Strategies to Improve Nursing Care for Monolingual Patients

Nurses emphasized the importance of enhancing effective communication to improve patient care and suggested strategies.

#### Subtheme 4.1: Recruitment of Trained Hospital Interpreters

Nurses have revealed that different people were interpreters during nursing care provision. However, the hospital did not employ them for this purpose, nor did they have formal training in language interpretation and translation. Thus, trained interpreters should be recruited to facilitate effective communication. Participant 7 suggested this “Maybe the government can create positions for professional translators” **N7**. Participant 6 added, “They can hire professional multilingual persons to be translators for the hospital” **N6**.

#### Subtheme 4.2: Incorporate Language Courses Into Nursing Curricula

Furthermore, nurses advocated for including language courses in the nursing curricula, emphasizing the need to provide local language foundation skills. To support this, Participant 6 mentioned that “the training institutions should introduce one or two language modules in the basic curriculum, whereby students can learn the basics before working in the health care industry. It will make it easier for nurses to communicate with patients effectively” **N6**.

#### Subtheme 4.3: Nurses’ In-Service Training on Local Languages

Regarding continuous professional development, nurses emphasized the importance of in-service training so that health professionals can be equipped with basic health care-related concepts in local languages. This way, they can comprehend patients’ health concerns and communicate effectively. Participant 6 further stated that, “we need continuous training whereby we learn different languages” **N6**. To emphasize the need for language training, participant 19 mentioned that, “there are a lot of training offered, but none include an introduction to languages, it will be better if we have sessions on languages so we can comfortably communicate with patients” **N19**.

#### Subtheme 4.4: Recruitment of Multilingual Nurses

In addition, a suggestion was made to prioritize the recruitment of multilingual nurses. Having staff who speak various languages could greatly enhance communication during the provision of nursing care. Participant 2 suggested, “Perhaps we can have other nurses who speak more than two languages, or when recruiting for each unit, consider one or two multilingual people before others” **N2**.

## Discussion

This qualitative study explored nurses’ experiences of providing nursing care to monolingual patients at a national referral hospital in Namibia. The study revealed that nurses benefited from the experience with monolingual patients by becoming culturally competent; they developed a desire to learn new languages and enhance nonverbal communication skills. In the United States, Ian et al. (2016) reported that nurses who worked with non-English speaking patients had increased awareness of patients’ needs, cultural competence, and personal development, which is comparable to the experiences of nurses in this study. Learning a new language as part of a nurse’s benefits enhances career development and the ability to study abroad (Dos Santos, 2021). This study further revealed that nurses use nonverbal communication techniques, such as pointing and facial expressions. Nonverbal communication is vital to patient-centered outcomes (Sharkiya, 2023). It involves passing information via bodily signals, including gestures, facial expressions, eye contact, and paralinguistics (vocal cues) (Aziz et al., 2021). The meanings of some nonverbal communication acts, such as touch, eye gaze, and head nodding, may be interpreted differently across cultures, which could hinder the interactions between nurses and patients (Kwame & Petrucka, 2021). The current result on nonverbal communication techniques during nursing care is not surprising. Due to a multilingual society, these practices are used to communicate in many encounters, such as Namibia’s public offices, shops, and other public places.

On negative experiences, nurses in this study revealed a language barrier when caring for monolingual patients. This led to delayed care delivery as nurses tried to look for interpreters or find alternative approaches to translate communication with the patient. Recent scoping reviews by Gerchow et al. (2021) and Abraham et al. (2024) revealed that nurses in Africa and globally face everyday complexities during care provision due to language barriers. Henceforth, this leads to a compromise of patient safety (Organi et al., 2024). In Saudi Arabia, Alhamami (2022) reported health care workers’ negative experiences of language discordance, such as a waste of time and resources to get interpreters and concerns about patients’ confidentiality and privacy. Although not openly revealed in this study, language barriers between patients and providers lead to wrong diagnoses, treatment errors, and frequent misunderstandings (Organi et al., 2024). Since there is a language barrier between monolingual patients and nurses, conducting proper patient assessments is difficult. This causes service providers to feel frustrated and low in self-confidence since they cannot fully assist the patients (Mustafa et al., 2023).

This study indicated that nurses use *ad hoc* interpreters to communicate with monolingual patients. In the provision of nursing care, interpreters ensure a sufficient flow of information to the patients in case of limited language skills (Heath et al., 2023; Probst & Imhof, 2016). Using *Ad hoc* interpreters is common in multilingual societies, as there is always a person among service recipients or clients who can interpret communication between service providers and other clients. Therefore, these results were not surprising.

Nurses in this study utilize mobile translation technology when providing nursing care to monolingual patients. Mobile translation can serve as an inexpensive alternative to professional translation, given its convenience and the fact that some applications do not require an internet connection, making it suitable for rural areas. To support this study’s findings, Chang and Maluda (2021) reported that nurses could explain prescribed medications and respond better to patient requests with the assistance of mobile translation services. van Vuuren et al. (2021) indicated that using mobile applications designed for the medical field helps convey terminology accurately when a language barrier is experienced. However, live mobile translation in this study setting is used minimally due to the unavailability of Namibian native languages on common platforms like “Google Translate.” It was more used for patients who only communicate in languages like Afrikaans and Portuguese. For native languages, pre-recorded information on mobile devices was reported to be used by nurses in this study.

Nurses in this study resorted to using simplified and plain language when providing care to monolingual patients. This approach facilitates communication when translation services or interpreters are unavailable. Resulting in building trust, stronger patient–provider relationships, effective therapeutic communication, patient satisfaction, comprehensive care, and culturally congruent care (Khattak et al., 2024). The reason for using simplified and plain language in this study is to help patients understand and comprehend what is communicated, and to follow instructions given, rather than using jargon and complex language.

Implicated by the multilingual nature of the society in this study setting, nurses suggested the recruitment of trained or professional interpreters. Similarly, van Vuuren et al. (2021) and Organi et al. (2024) supported using professional interpreters in a multicultural society. Compared with other types of interpretation or none, professional interpreters are associated with the greatest satisfaction and best patient communication (Heath et al., 2023). However, the recruitment process should exercise coverage and equity regarding the number of native languages spoken. Squires (2018) advises that for proper professional translation, staff members who speak other languages fluently should be assisted in obtaining medical interpreter training and certification for their skills.

Moreover, another way to improve nursing care for monolingual patients is to incorporate language courses into nursing curricula. This would enhance nurses’ ability to speak more languages, which could be a facilitating factor in acquiring cultural competence (Matthews & Van Wyk, 2018). Adding languages may be suggested as an extracurricular activity or a noncredit-bearing course to avoid extra credit.

Another strategy proposed to improve nursing care for multilingual patients is recruiting multilingual nurses. While local and international regulations on recruiting nurses call for “freedom from discrimination” (International Council of Nurses [ICN], 2019, p. 3), considering multilingual nurses, among others, during the recruitment process was seen as an initiative to ensure adequate coverage of native languages. Similarly, a previous Namibian study on nursing students supported recruiting multilingually fluent nurses (Ashipala & Matundu, 2023). Furthermore, this study calls for nurses’ in-service training in local languages. This is because they lack the basic linguistic skills necessary to communicate with patients who do not speak the same languages as them. In-service training in health care settings is usually conducted through workshops, seminars, and on-the-spot training. Training and education on language and cultural competence are needed to handle diverse patients (Gerchow et al., 2021; Organi et al., 2024). Providing supportive initiatives for nurses’ language skills, and implementing them in nursing care are important in reducing problems arising from language barriers (Hemberg & Sved, 2021).

Our study provided new insightful findings that nurses develop cultural competence and a desire to learn new languages through the nursing of monolingual patients, and their interactions with patients enhance nonverbal communication skills. However, patients’ privacy was compromised, and it was difficult to maintain confidentiality. Moreover, a unique, insightful finding was that strategies to improve care include incorporating local language courses into the nursing curriculum and the nurses’ in-service training program. The in-service training for nurses in local languages is highly supported; therefore, hospitals may hire language experts to conduct training or request training institutions to design short courses for health professionals.

### Limitations

The data mainly came from female nurses due to the limited number of males who work in the hospital departments where the study was conducted. Moreover, more participants indicated *Oshiwambo* as their mother tongue; however, the authors have no control over this, as it is the most commonly spoken native language in Namibia. These demographic characteristics might have influenced the nurses’ experiences.

## Conclusion and Recommendations

By exploring the experiences of caring for monolingual patients through the lens of nurses in a multicultural society, this study concluded that nurses get personal benefits while having negative experiences. The study offered alternative communication methods and strategies that could be employed to enhance nursing care for monolingual patients. These findings have implications for offering culturally congruent care. The findings emphasize the need to recruit professional interpreters and multilingual nurses, and incorporate languages into undergraduate curricula and in-service training for nurses. Therefore, the findings may be utilized to develop implementation guidelines for caring for monolingual patients in multicultural and multilingual societies, specifically to address language barriers and foster communicative competencies among nurses. As recommendations, nurses should conduct informal educational sessions on languages and cultural issues, and have common phrases and instructions written in different languages. In addition, when drafting duty rosters and delegating duties, consider mixing nurses according to their language proficiency. Future researchers may explore monolingual patients’ perceptions of interpreter-mediated nursing care. In addition, further studies on the experiences of ad hoc interpreting and interventions to enhance the professionalization of the interpreting workforce are required.
